# Supplementary material for: The eukaryotic-like characteristics of small GTPase, roadblock and TRAPPC3 proteins from Asgard archaea
Source: Commun Biol. 2024 Mar 12;7:273. doi: 10.1038/s42003-024-05888-1 (PMC10933270; doi:10.1038/s42003-024-05888-1)
Supplement: Supplementary file 2 — Supplementary Information [file 42003_2024_5888_MOESM2_ESM.pdf]

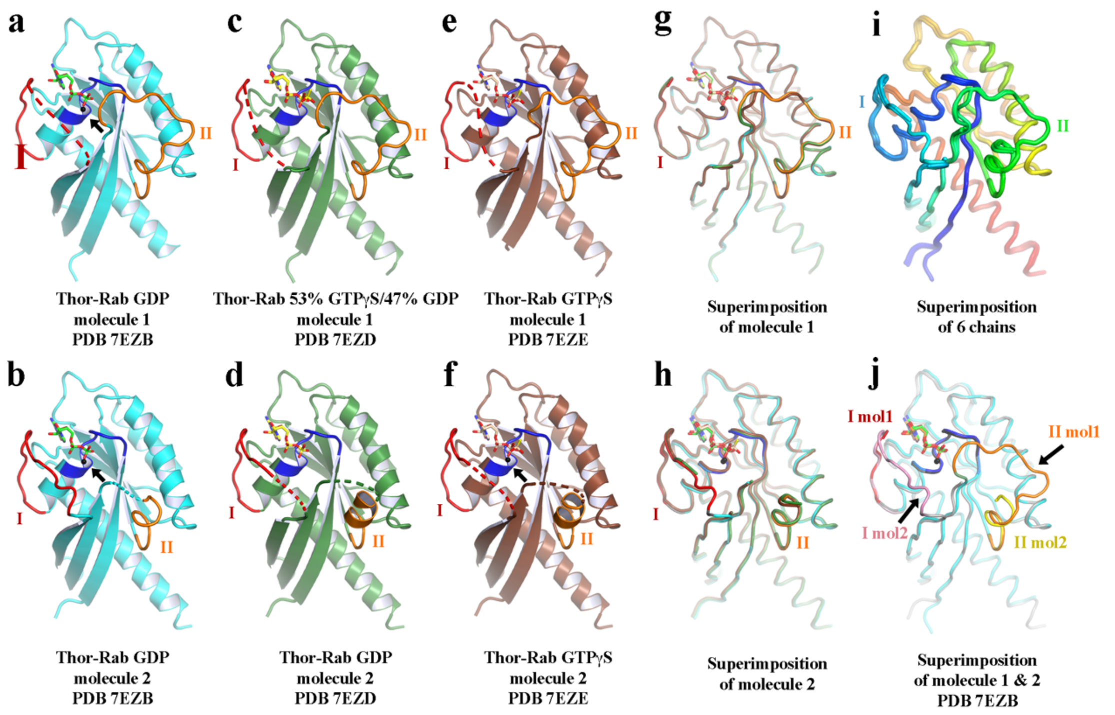

Supplementary Figure 1. The structure of Thor-Rab. (**a-f**) Comparison of the structures of Thor-Rab bound to different nucleotides from the two copies within the crystallographic asymmetric unit. The P-loop, Switch I and Switch II regions are colored blue, red and orange, respectively. The nucleotides are shown as sticks and magnesium cations as black spheres, highlighted by arrows. (**g-h**) Superimposition of the copies of molecule 1 or molecule 2, respectively. 7EZB traces are colored as in (**a**,**b**). 7EZD and 7EZE chains are colored as green and brown, respectively. (**i**) The rainbow trace representation shows the superimposition of the six chains. Blue to red, N- to C-termini. (**j**) Superimposition of the GDP-bound molecule 1 (cyan) and molecule 2 (gray) from 7EZB. Switch I (I) and Switch II (II) regions are colored red and orange, or pink and yellow, from molecule 1 and molecule 2, respectively

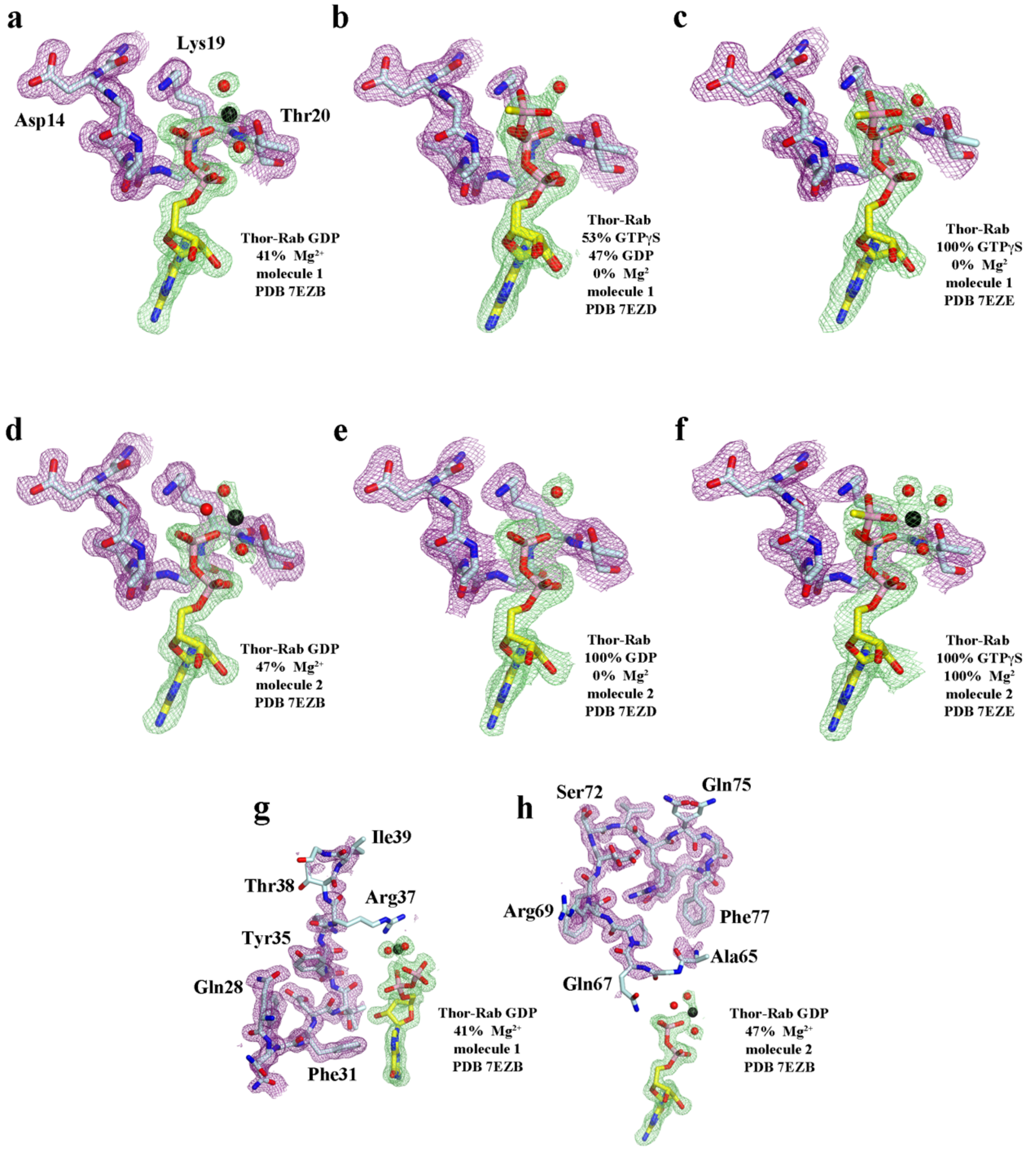

Supplementary Figure 2. Electron density around the nucleotide-binding site in Thor-Rab. (**a-e**) The OMIT electron density maps contoured at 1σ. Electron density surrounding the nucleotides and cations is shown as a green mesh. Electron density surrounding protein chain is shown as a purple mesh. Protein chains and nucleotide carbon atoms are shown in light blue and yellow, respectively. Mg^{2+} and waters are shown as black and red spheres, respectively. The average Mg^{2+} coordination distances are 2.22 Å, 2.29 Å and 2.19 Å in **a**, **d** and **f**, respectively. Selected residues are labelled. (**g**) Electron density surrounding the Switch I loop. There is no electron density for residue Arg37 (red triangle in Fig. 1d). (**h**) Electron density surrounding the Switch II loop. There is no electron density for residue Gln67 (orange triangle in Fig. 1d).

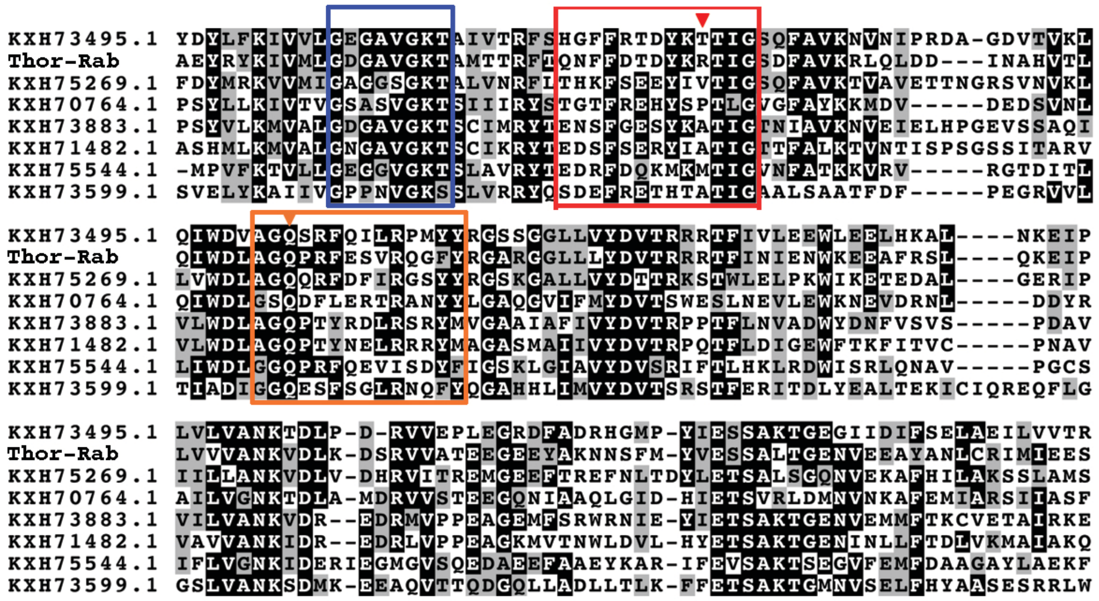

Supplementary Figure 3. A sequence alignment of the GTPase domain from closely related Thor-Rab paralogs. Switch I (red),Switch II (orange), P-loop (blue) sequences are highlighted by boxes. Triangles indicate the position of the two residues implicated in the catalytic mechanism for MglA. The Switch I arginine in Thor-Rab is not present in the other Thor-Rab paralogs, implying that they may require GAPs to enhance hydrolysis.

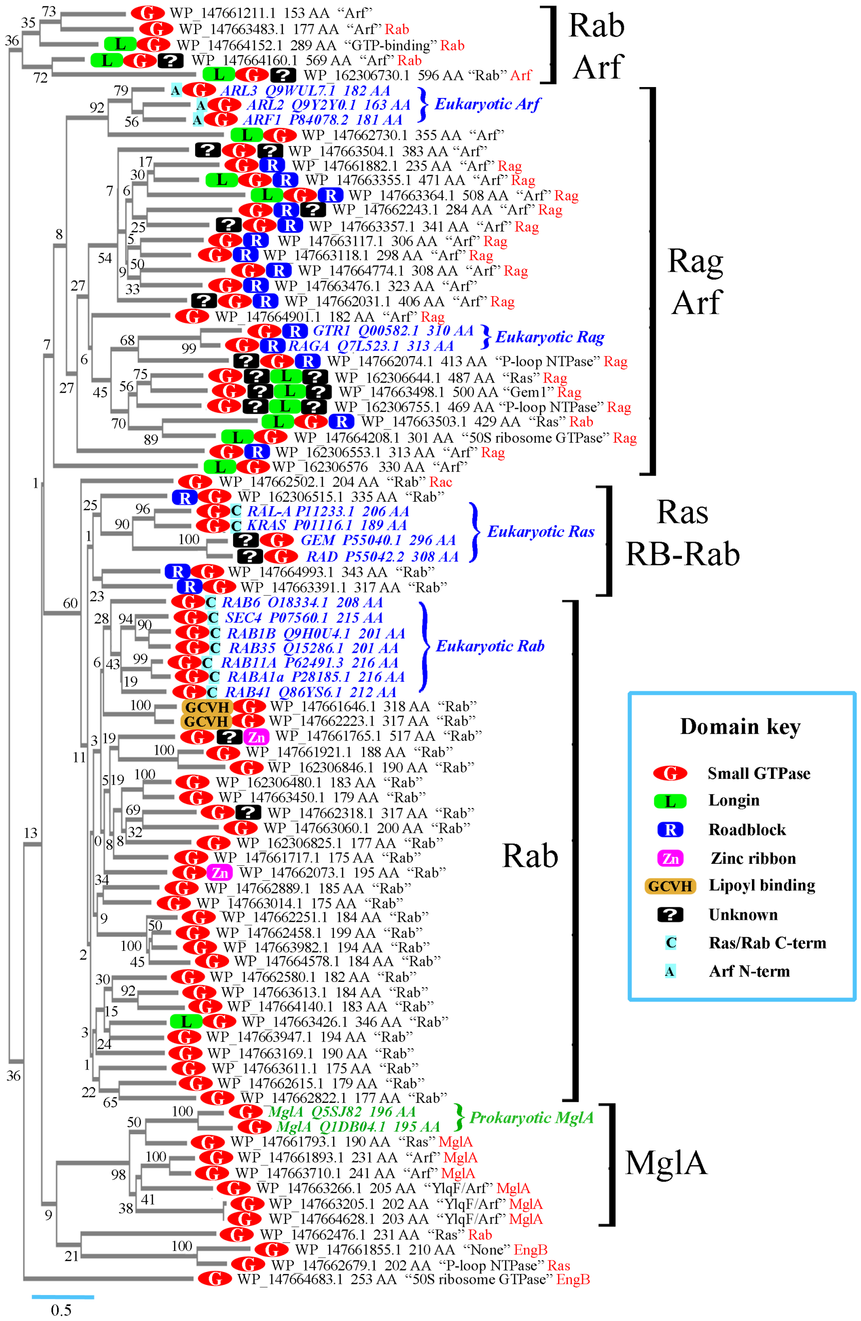

Supplementary Figure 4. A phylogenetic tree comparing MKD1 small GTPases to eukaryotic (blue) and prokaryotic (green) small GTPases. The tree was calculated on a structure-based sequence alignment, using structures for all the prokaryotic and eukaryotic sequences and Thor-Rab. Domain architectures, predicted by AF2, for the MKD1 sequences are shown as cartoons. Each protein is labelled with the NCBI accession number, the number of amino acids (AA), the NCBI accession definition in quotation marks, and the definition of the most similar structure from PDBeFOLD (red) if it differs from the NCBI accession definition. MKD1 GTPase sequences that are not included in this analysis are listed in (Supplementary Table 3).

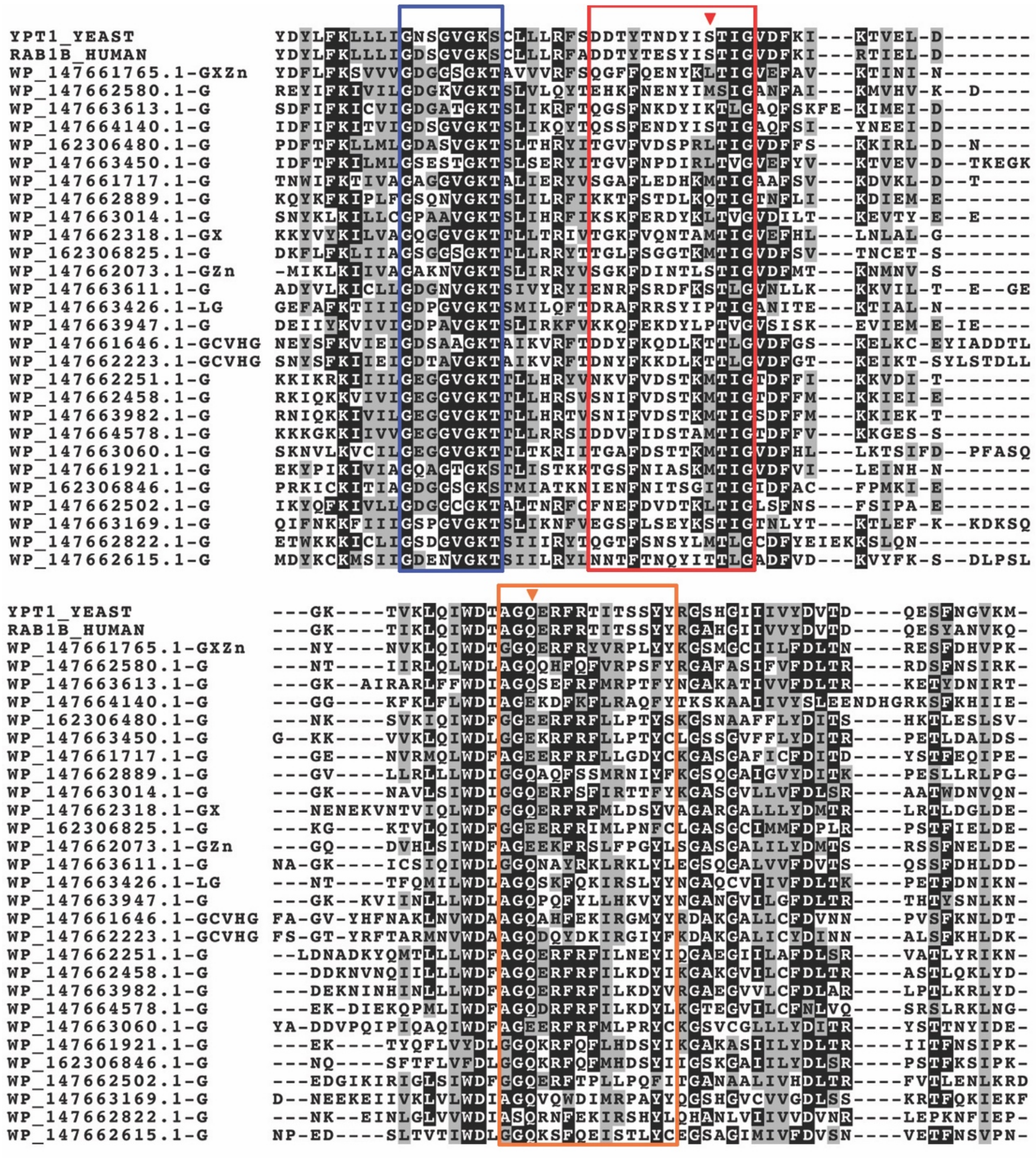

Supplementary Figure 5.

Supplementary Figure 5 (continued)

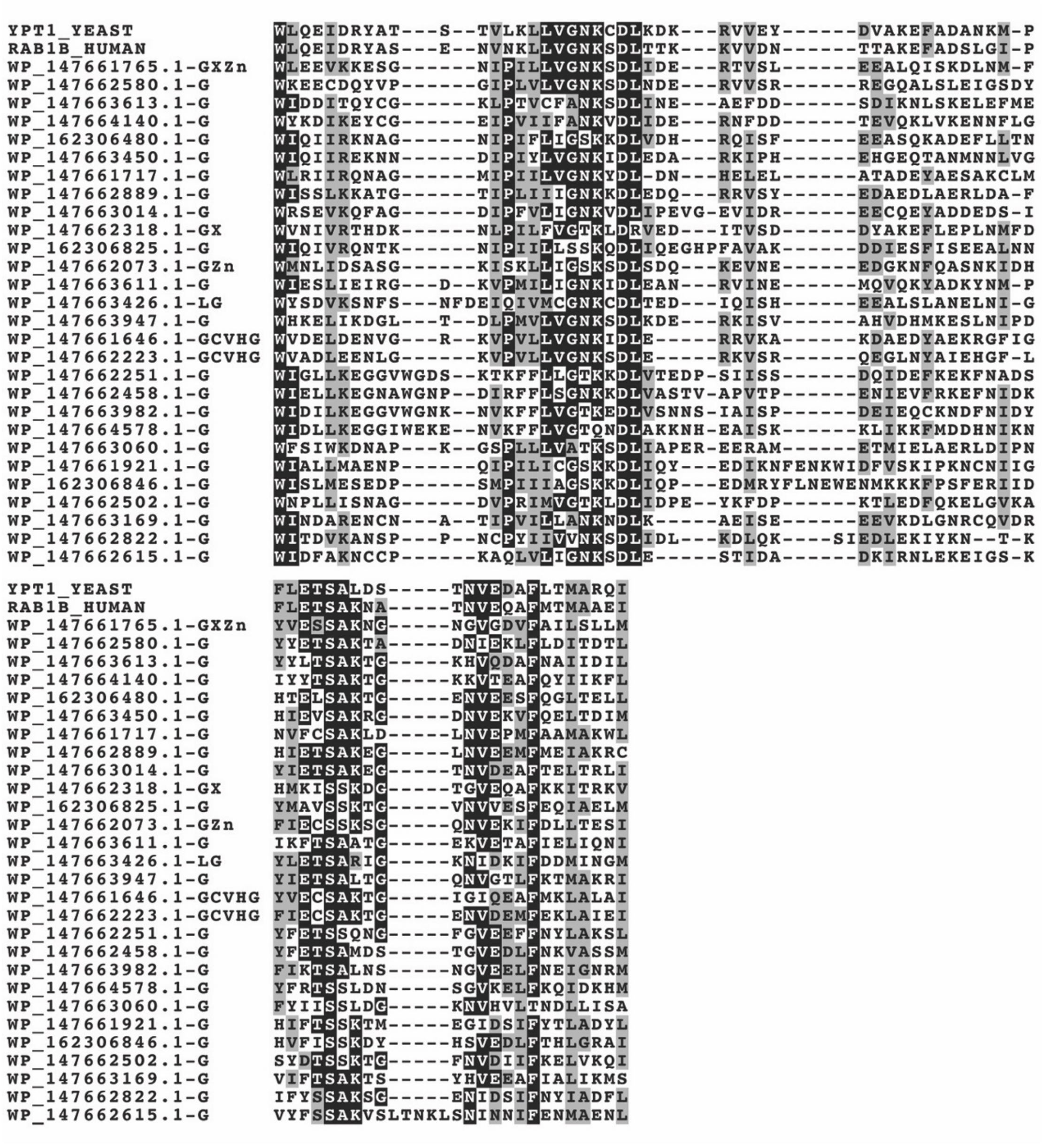

Supplementary Figure 5. A sequence alignment of the GTPase domain of MKD1 Rab paralogs compared to eukaryotic Rab sequences. GTPase sequences from the "Rab" clade from the phylogenetic tree in Supplementary Figure 4 are aligned. Switch I (red), Switch II (orange), P-loop (blue) sequences are highlighted by boxes. Triangles indicate the two residues implicated in the catalytic mechanism for MglA. The Switch I arginine in is not present in the MKD1 Rab paralogs, implying that they may require GAPs to enhance hydrolysis. Letters after the accession numbers indicate domains, as in the Supplementary Figure 4 key.

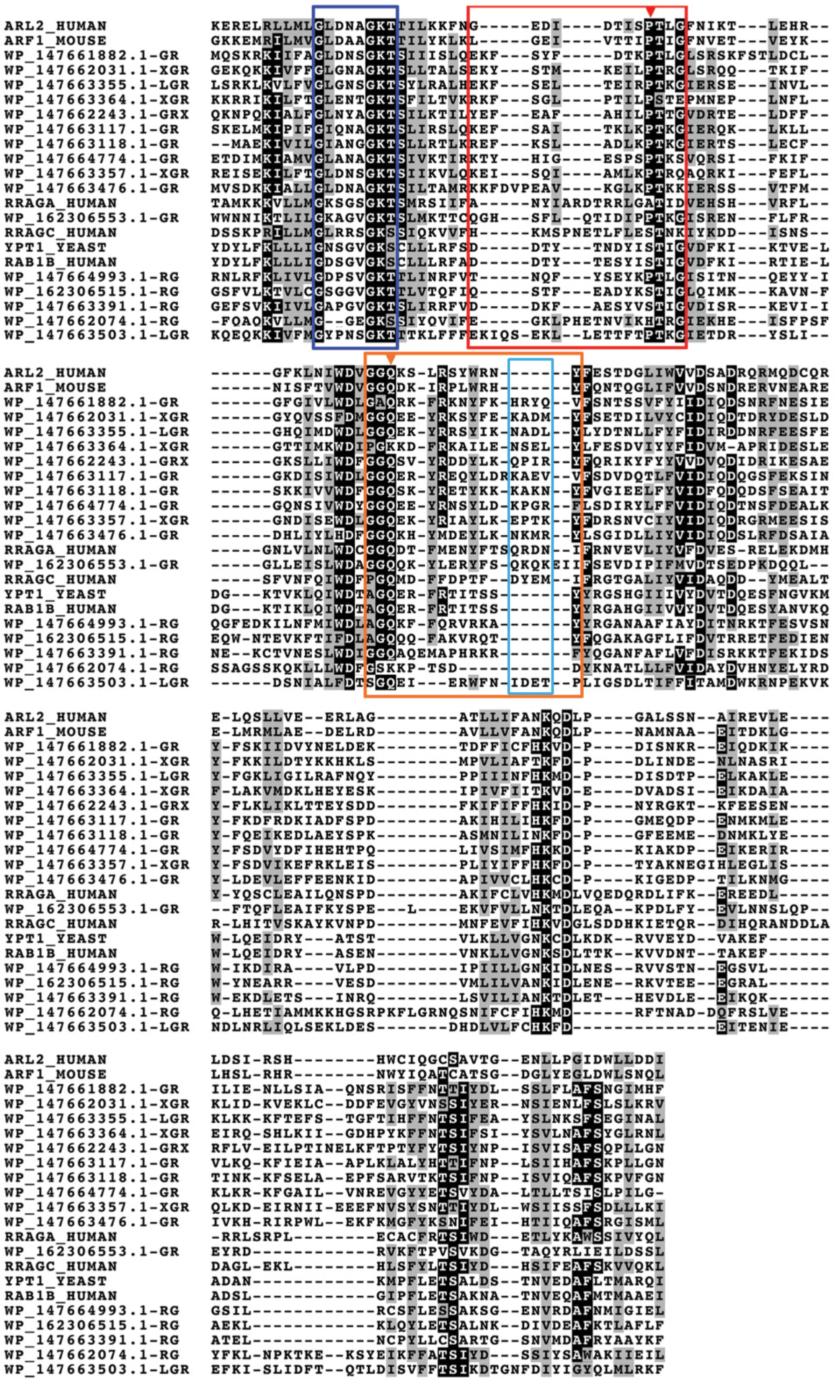

Supplementary Figure 6. (a) A sequence alignment of the GTPase domains of MKD1 from RB domain-containing paralogs, compared to eukaryotic Rag/ARF sequences. Switch I (red), Switch II (orange), P-loop (blue) sequences are highlighted by boxes. Triangles indicate the two residues implicated in the catalytic mechanism for MglA. The Switch I arginine in is not present in the MKD1 RB GTPase paralogs, implying that they may require GAPs to enhance hydrolysis. Letters after the accession numbers indicate domains, as in the Supplementary Figure 4 key. The cyan box indicates an insert in Switch II present in “GR” but not in “RG” architectures.

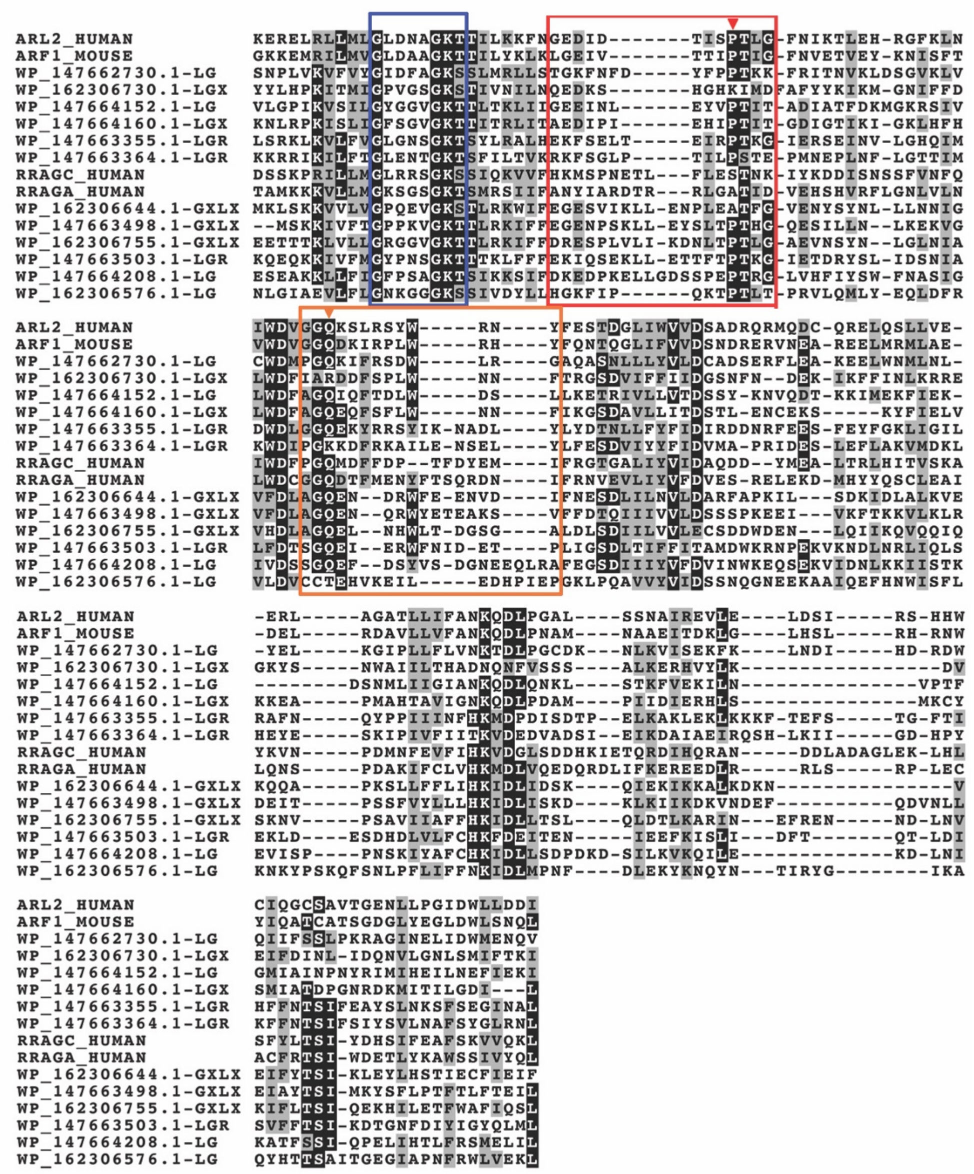

Supplementary Figure 6 (continued). (b) A sequence alignment of the GTPase domains of MKD1 from longin domain-containing paralogs, compared to eukaryotic Rag/ARF sequences. Switch I (red), Switch II (orange), P-loop (blue) sequences are highlighted by boxes. Triangles indicate the two residues implicated in the catalytic mechanism for MglA. The Switch I arginine in is not present in the MKD1 longin GTPase paralogs, implying that they may require GAPs to enhance hydrolysis. Letters after the accession numbers indicate domains, as in the Supplementary Figure 4 key.

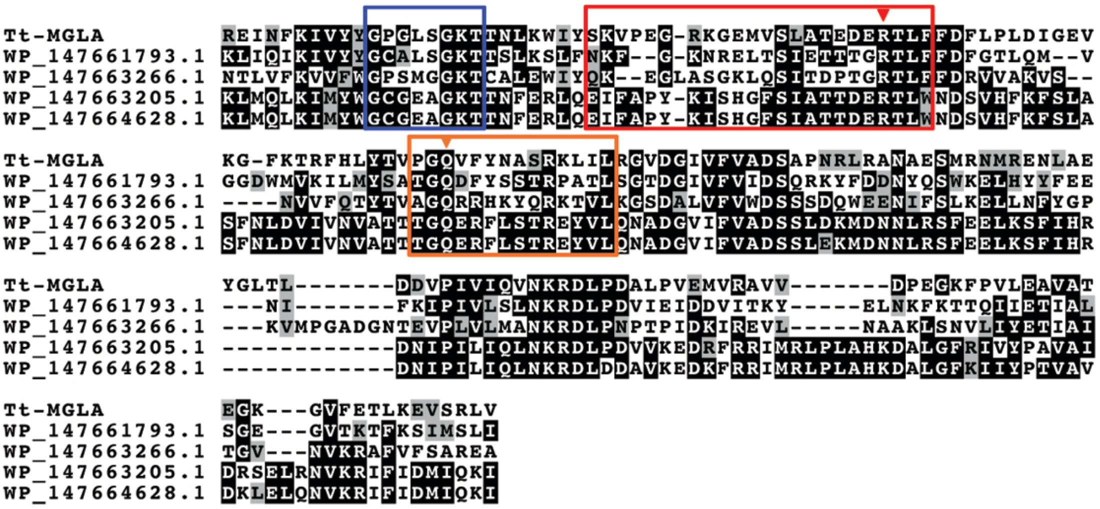

Supplementary Figure 7. A sequence alignment of the GTPase domain of MKD1 MglA paralogs compared to a bacterial MglA sequence. Switch I (red), Switch II (orange), P-loop (blue) sequences are highlighted by boxes. Triangles indicate the two residues implicated in the catalytic mechanism for MglA. The Switch I arginine and Switch II glutamate are conserved in the MKD1 MglA paralogs, implying that they may not require GAPs to enhance hydrolysis, and can achieve this through allosteric mechanisms. Similarly to bacterial *Thermus thermophiles* MglA, Switch I in the MKD1 MglA paralogs is expanded compared to the MKD1 Rab paralogs (Supplementary Fig. 5).

Supplementary data

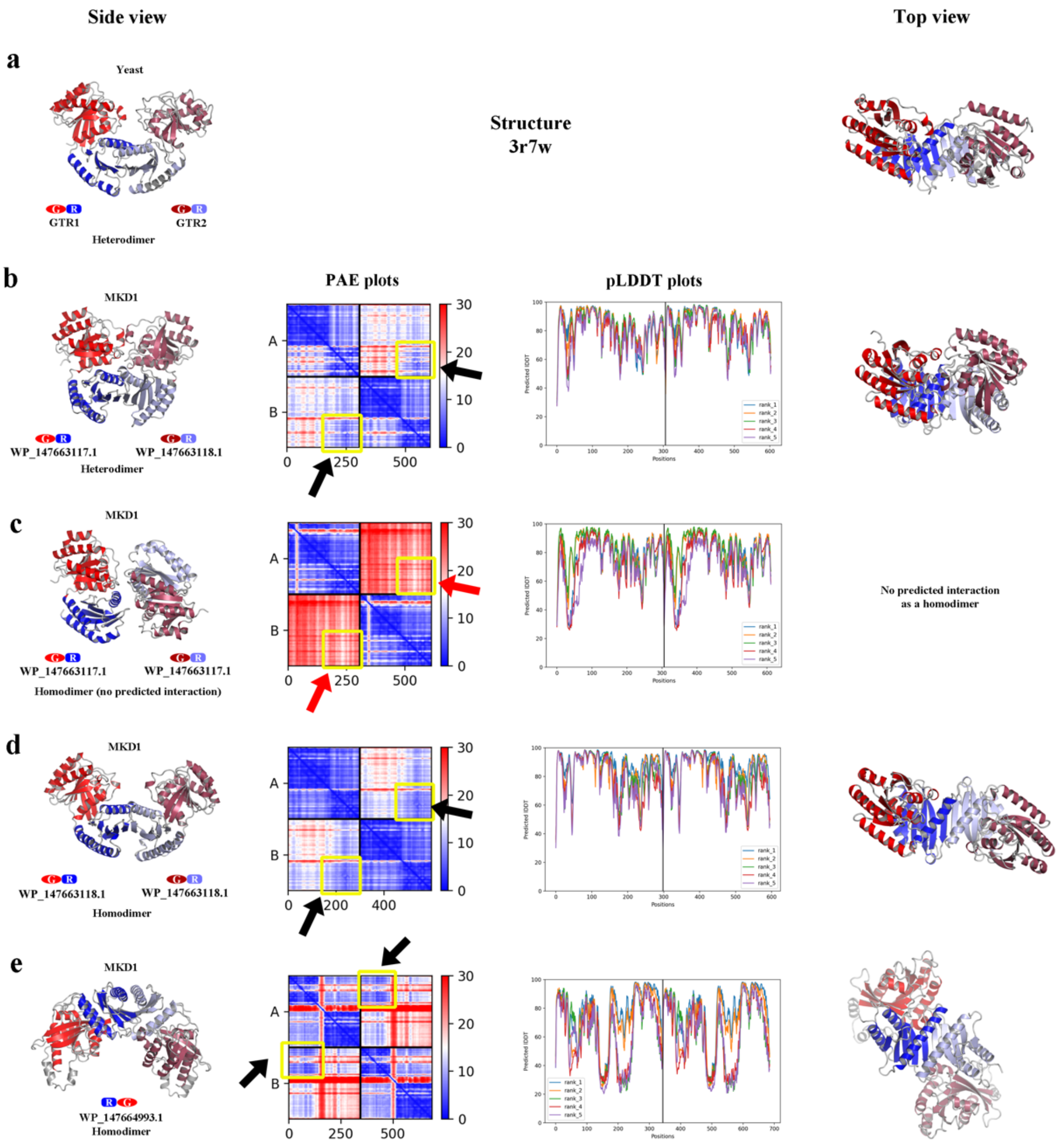

Supplementary Figure 8.

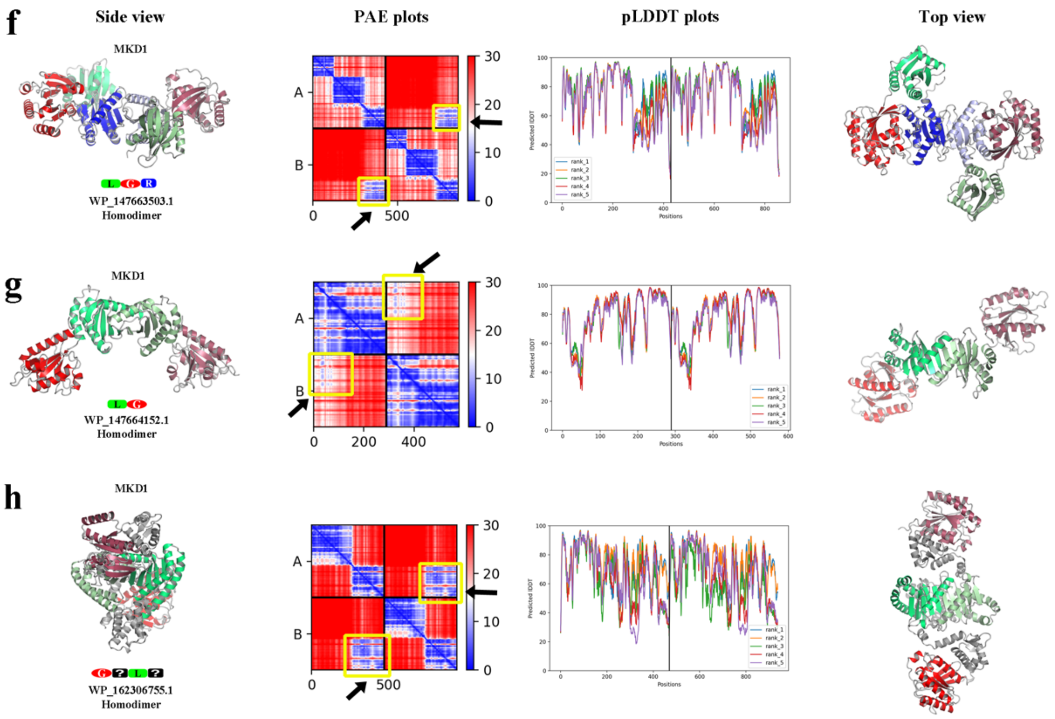

Supplementary Figure 8 (continued). Representative AF2 predictions as dimeric proteins for MKD1 GTPase protein that contain RB or longin domains. The predicted structures are colored as in the cartoons of the domain organization, with slightly different hues for the second copy of the dimers. The dimers are shown in two orientations to highlight the positions GTPase domain relative to the longin or RB domains. (**a**) The crystal structure of the EGO complex Rag-like GTPase heterodimer from yeast (PDB ID 3R7W) for comparison. (**b-d**) AF2 predicted structures of combinations of the two Rag-like GTPases from a single operon in the MKD1 genome. One homodimer (**c**) is not predicted to exist. (**e-h**) Predictions of longin-domain containing GTPases as homodimers. With the exception of (**c**), AF2 predicts typical RB or longin domain dimeric interactions. The central panels show the PAE and pLDDT plots. The intermolecular interactions are highlighted by yellow boxes and black arrows in the PAE plots, which in (**g**) indicates a low confidence interaction in this region, whilst (**c**) shows no interaction. Domain codes: G, GTPase; R, roadblock domain; L, longin domain; "?", various domains of unknown function.

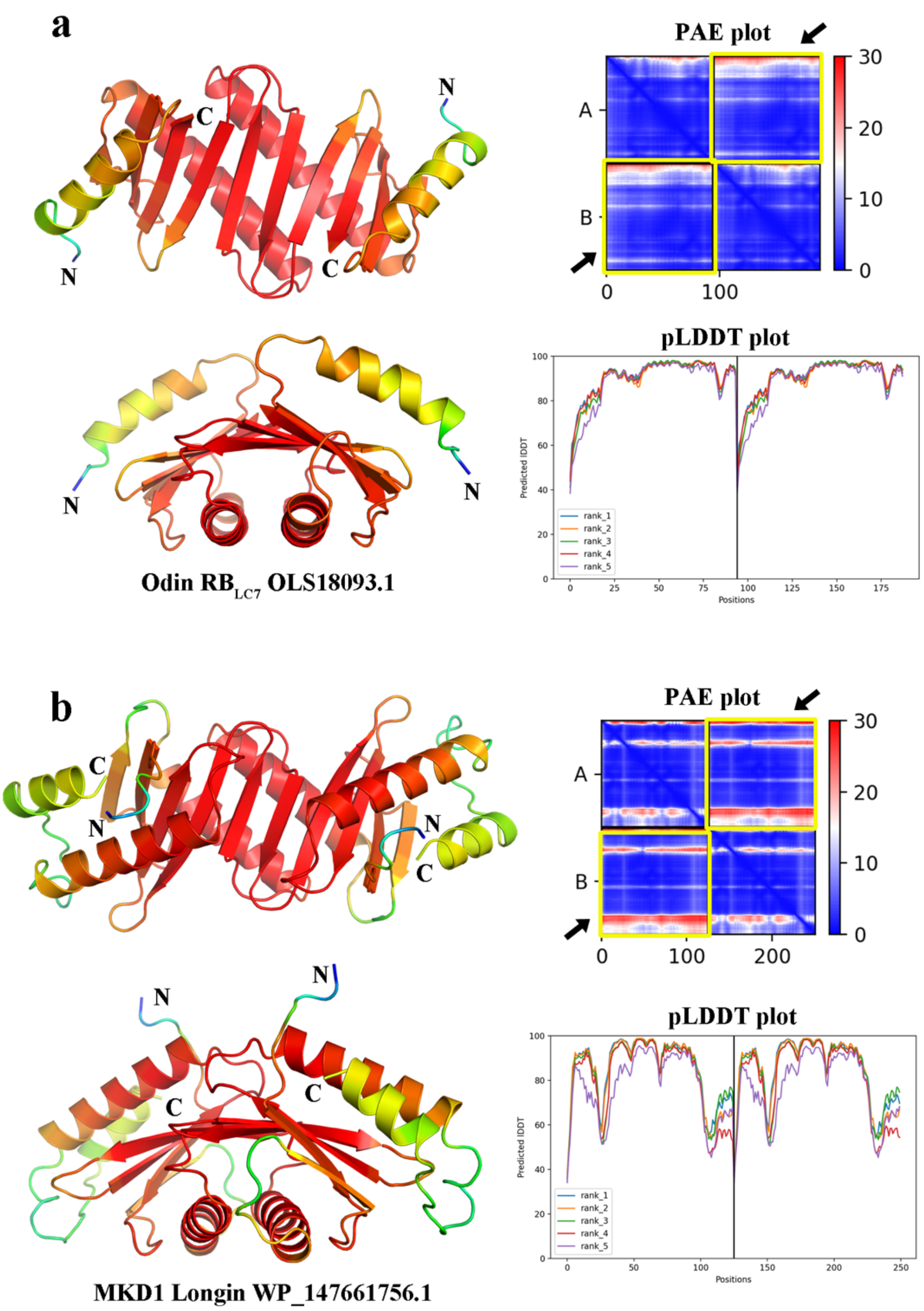

Supplementary Figure 9. Two views of the AF2-predicted structures of (a) Odin-RB$_{LC7}$ homodimer and (b) MKD1 longin homodimer. Red-to-blue spectrum indicates high to low confidence levels. The MKD1 longin model is most similar to the mouse longin protein TRAPPC1 (Bet5) structure from PDB entry 2J3T, characterised by 111 aligned amino acids with RMSD of 2.2 Å. The PAE and pLDDT plots are shown. The intermolecular interactions are highlighted by yellow boxes and black arrows in the PAE plots, indicating confidence within the homodimer interactions in the predictions.

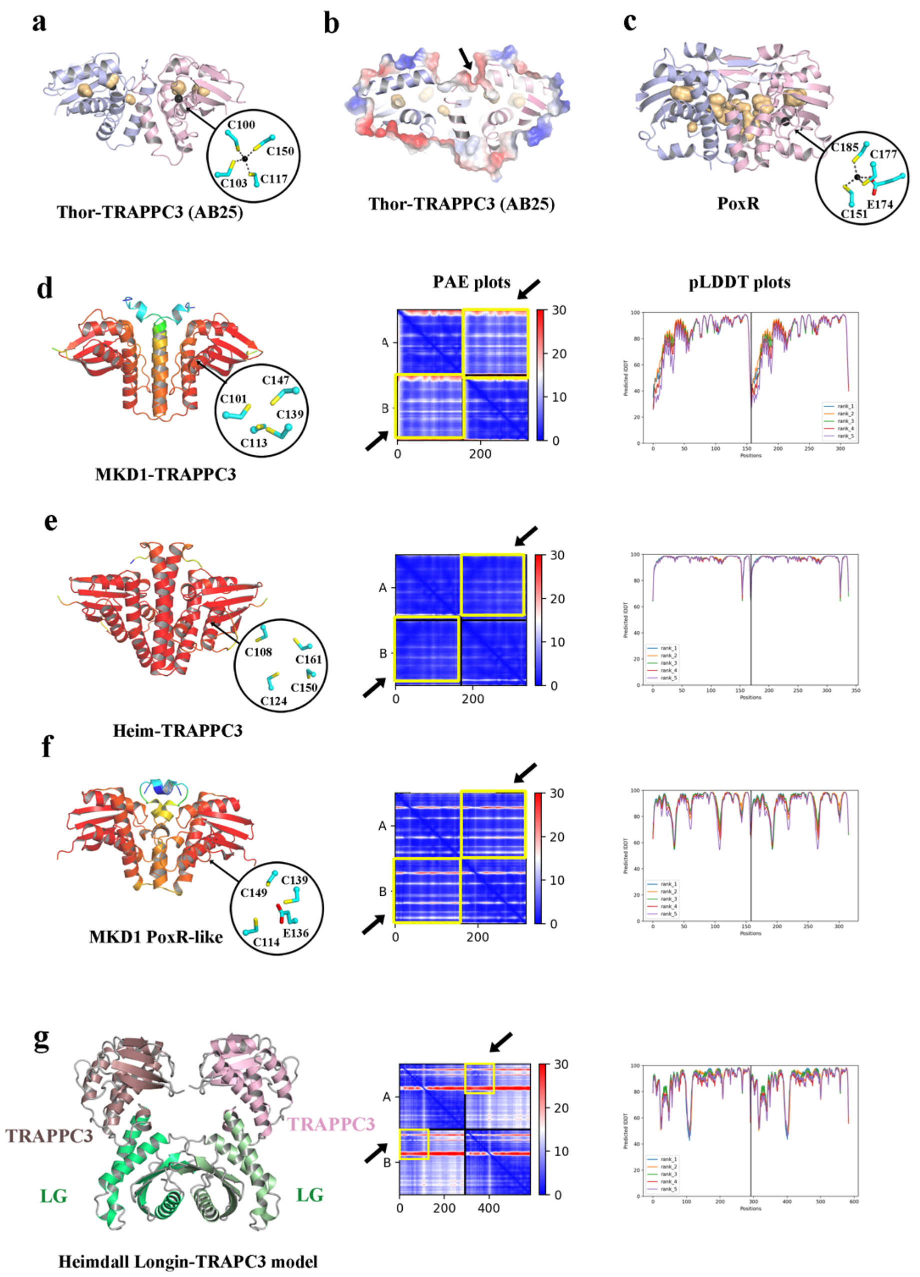

Supplementary Figure 10. Comparison of Thor-TRAPPC3 with other TRAPPC3/V4R structures. (**a**,**b**) The structure of the Thor-TRAPPC3 (AB25) homodimer for comparison with Fig. 8. (**c**) The entire V4R domain from PoxR (PDB ID 5FS0). (**d-f**) AF2 models of potential TRAPPC3 and V4R domain proteins from MKD1 (Loki) and Heimdall. Red-to-blue spectrum indicates high to low confidence levels. Circles indicate potential, or experimentally determined Zn^{2+}-binding sites. (**g**) AF2 predicted model of a longin-TRAPPC3 homodimer. The PAE and pLDDT plots are shown. The intermolecular interactions are highlighted by yellow boxes and black arrows in the PAE plots, indicating confidence within the homodimer interactions in the predictions.

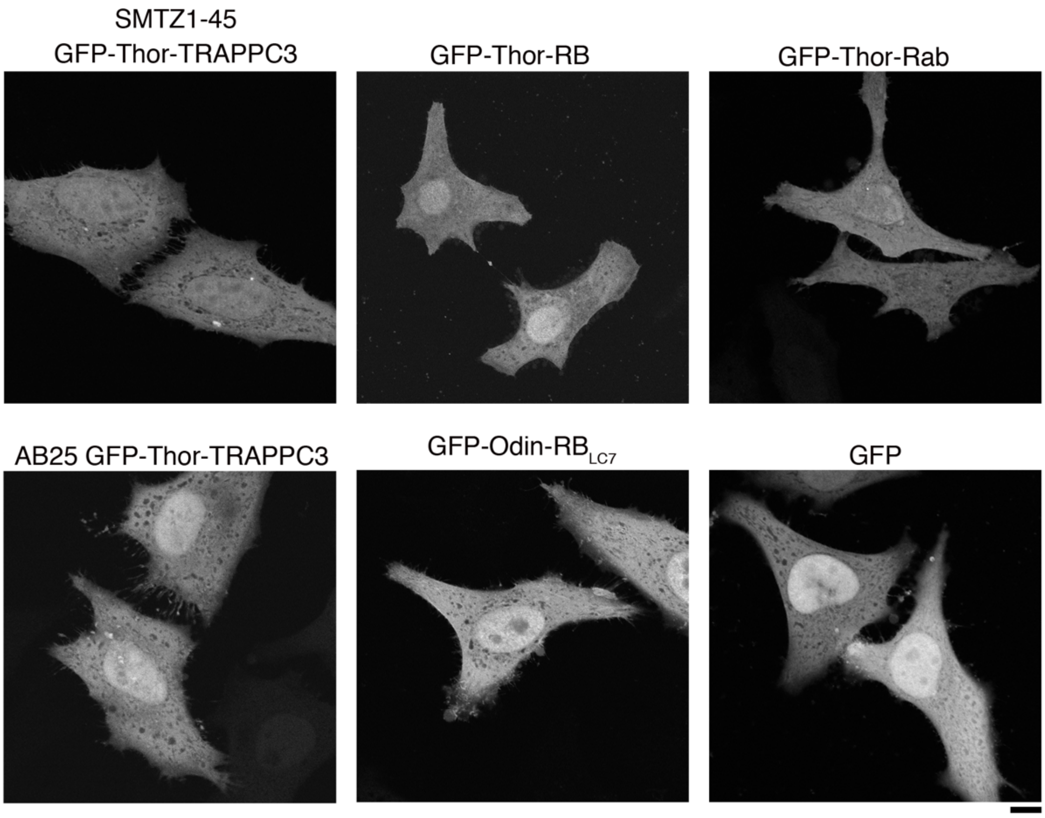

Supplementary Figure 11. Localization of Asgard proteins on heterologous expression in human HeLa cells. No distinct membrane localization was observed. Scale bar = 10 μm.

Supplementary Table 1. X-ray data collection and refinement statistics for Thor-Rab.

	Thor-Rab GDP Thor (SMTZ1-45) KXH73347.1 (PDB code 7EZB)	Thor-Rab GTPγS Thor (SMTZ1-45) KXH73347.1 (PDB code 7EZD)	Thor-Rab GTPγS/Mg^{2+} (soak) Thor (SMTZ1-45) KXH73347.1 (PDB code 7EZE)
**Data collection**			
Beamline	TPS 05A, NSRRC	BL41XU, SPring-8	TPS 05A, NSRRC
Crystallization conditions	100 mM MES, pH 5.0 200 mM NaCl 13% PEG3350 5 mM GTP/5 mM GDP	100 mM MES, pH 5.0 200 mM NaCl 13% PEG3350 10 mM GTPγS	100 mM MES, pH 5.0 200 mM NaCl 13% PEG3350 5 mM GTP/5 mM GDP
Soak	None	None	10 mM GTPγS 15 mM $MgCl_2$
Crystal	$P3_1$	$P3_1$	$P3_1$
a, *b*, *c* (Å)	68.8, 68.8, 66.3	68.7, 68.7, 66.6	69.1, 69.1, 66.9
α, β, γ (°)	90.0, 90.0, 120.0	90.0, 90.0, 120.0	90.0, 90.0, 120.0
Wavelength (Å)	1.0	1.0	1.0
Resolution (Å)	20.0-1.50 (1.53-1.50)	44.4-1.76 (1.79-1.76)	20.0-1.95 (1.98-1.95)
R_{merge}	10.3 (101.2)	7.8 (112.3)	8.7 (82.0)
R_{meas}	11.4 (117.5)	8.7 (125.2)	9.6 (96.4)
R_{pim}	4.9 (58.9)	3.8 (54.9)	4.1 (49.8)
$I/\sigma(I)$	18.9 (1.9)	11.0 (1.5)	16.7 (1.2)
$CC_{1/2}$	(0.489)	(0.558)	(0.507)
Completeness (%)	100.0 (100.0)	99.9 (98.4)	97.8 (99.5)
Redundancy	5.4 (3.8)	5.2 (5.2)	5.4 (3.5)
**Refinement**			
Resolution (Å)	17.2-1.5 (1.55-1.50)	44.4-1.76 (1.82-1.76)	20.0-1.95 (2.02-1.95)
No. reflections	55400 (4850)	34772 (3430)	25490 (2590)
R_{work} / R_{free}	15.6/17.4 (22.7/23.1)	16.4/18.9 (25.0/26.0)	16.6/20.1 (25.6/29.4)
No. atoms			
Protein	2941	2938	2845
Ligand	58	88	65
Nucleotide in molecule A	100% GDP	100% GDP	100% GTPγS
Nucleotide in molecule B	100% GDP	53% GTPγS, 47% GDP	100% GTPγS
Occupancy of Mg^{2+} site in molecule A	41%	0%	100%
Occupancy of Mg^{2+} site in molecule B	47%	0%	0%
Water	411	286	286
B factors			
Protein	22.2	33.0	33.6
Ligand	15.7	28.7	31.6
Water	34.8	41.2	41.9
r.m.s deviations			
Bond lengths (Å)	0.007	0.004	0.003
Bond angles (°)	0.95	0.75	0.64
Ramachandran Plot			
Favoured (%)	98.5	100.0	98.5
Outliers (%)	0.0	0.0	0.3

Supplementary Table 2. Structural similarity of Thor-Rab with other small GTPases.

						Thor-Rab		
	Family	Species	PDB	Nucleotide	Res/Å	RMSD/Å	Residues	Rank
**RAB8A**	Rab	Human	6stf:E	GDP/Mg^{2+}	2.4	1.1	147	1
**KRAS**	Ras	Human	6pgp:A	GDP/Ca^{2+}	1.5	1.0	145	4
**HRAS**	Ras	Human	3lo5:A	GDP/Ca^{2+}	2.6	1.0	141	5
**RAB1A**	Rab	Human	2fol:A	GDP/Mg^{2+}	2.6	1.1	145	6
**RABA1A**	Rab	Arabidopsis	5xr4:A	GDP/Mg^{2+}	2.8	1.3	145	12
**RalA**	Ras (Ral)	Human	2a9k:A	GDP/Mg^{2+}	1.7	1.1	146	70
**Rit1**	Ras (Rit)	Human	4klz:A	GDP/Mg^{2+}	2.3	1.0	141	263
**SEC4**	Rab	Yeast	1g16:A	GDP/Co^{2+}	1.8	1.1	142	279
**RHEB**	Ras (Rheb)	Human	7bta:B	GDP	2.6	1.3	141	346
**RAP1B**	Ras (Rap)	Human	6uzk:B	GNP/Mg^{2}	1.9	1.2	142	448
**RRAD**	Ras (Rad)	Human	2gjs:A	GDP/Mg^{2+}	1.9	1.4	140	585
**RhoA**	Rho	Human	5hpy:B	GDP/ Mg^{2+}	2.4	1.4	140	750
**Ran**	Ran	Human	1ibr:C	GNP/Mg^{2+}	2.3	1.2	138	893
**CDC42**	Rho	Human	5cjp:C	GTP/Mg^{2+}	2.6	1.4	139	904
**Rac1**	Rho	Human	1i4l:D	GTP/Mg^{2+}	2.7	1.3	139	932
**ARL1**	Arf	Human	4dcn:B	GTP/Mg^{2+}	3.0	2.1	136	1428
**MglA**	MglA	*Tt*	3t1o:A	GDP/Mg^{2+}	1.9	2.6	141	1435
**EngB**	EngB	*Tm*	3pqc:A	GDP	1.9	3.0	124	1939

Tt = Thermus thermophilus HB8, *Tm = Thermotoga maritima*, Res = Resolution

The closest structural matches to Thor-Rab. The relatedness is given as RMSD over the number of aligned residues. The rank is based on highest Dali Z-scores[1].

Supplementary Table 3. Sequences omitted from phylogenetic analysis (Supplementary Fig. 4)

Accession code	No. AA	Type of GTPase	Comment	Structure hit
WP_147661245.1	259	GPN-loop	Transcription/Translation	5hci
WP_147661298.1	353	TRAFAC NOG1	Translation	2e78
WP_162306528.1	424	TRAFAC HflX	Translation	3kxi
WP_147662421.1	318	TRAFAC DRG	Translation	4a9a
WP_147663500.1	378	TRAFAC DRG	Translation	4a9a
WP_147663179.1	163	Rab	P-loop motif missing	12il
WP_147662714.1	742	FEOB	Iron import	NS

Supplementary Table 4. X-ray data collection and refinement statistics for RB proteins.

	Odin-RB_{LC7}<br>Odin (LCB_4)<br>OLS18093.1<br>(PDB code 7F8F)	Thor-RB<br>Thor (SMTZ1-45)<br>KXH72322.1<br>(PDB code 7F8M)	MKD1-RB<br>*P. syntrophicum*<br>WP_147663254.1<br>(PDB code 7YH1)
**Crystals**			
Crystallization conditions	200 mM $MgSO_4$<br>20% PEG 4000<br>10% glycerol	100 mM CH_3COONa<br>pH 5.0, 200 mM LiCl<br>16% PEG 6000	100 mM MES, pH 6.0<br>250 mM $Zn(CH_3COO)_2$<br>6% PEG 3000
Lattice	$I2_12_12_1$	$P3_1$	P1
a, *b*, *c* (Å)	58.4 58.4 189.2	59.0, 59.0, 194.0	48.3, 53.6, 59.9
α, β, γ (°)	90.0, 90.0, 120.0	90.0, 90.0, 120.0	76.7, 75.3, 80.6
**Data collection**			
Beamline	TPS 05A, NSRRC	TPS 05A, NSRRC	TPS 05A, NSRRC
Wavelength (Å)	1.0	1.0	1.0
Resolution (Å)	42.1-1.83 (1.87-1.83)	20.0-2.14 (2.19-2.14)	20-2.69 (2.75-2.69)
R_{merge}	4.6 (52.4)	8.5 (59.9)	4.9 (18.5)
R_{meas}	5.0 (56.6)	9.5 (68.9)	6.9 (26.1)
R_{pim}	1.9 (21.1)	4.3 (33.2)	4.9 (18.5)
$I/\sigma(I)$	18.5 (2.6)	35.2 (2.3)	23.7 (4.4)
$CC_{1/2}$	(0.889)	(0.664)	(0.952)
Completeness (%)	99.8 (97.6)	99.0 (98.0)	91.6 (94.2)
Redundancy	7.1 (7.0)	4.8 (3.6)	1.9 (1.9)
**Refinement**			
Resolution (Å)	39.3-1.83 (2.10-1.83)	20.0-2.14 (2.20-2.14)	19.8-2.69 (2.79-2.69)
No. reflections	8417 (2619)	38418 (2219)	14119 (1384)
R_{work} / R_{free}	19.3/21.6 (20.1/22.0)	19.1/26.0 (26.5/30.8)	22.2/26.6 (33.0/35.9)
No. atoms			
Protein	639	5291	3612
Ligand	-	-	12 Zn^{2+}
Water	45	306	62
B factors			
Protein	31.7	34.3	66.0
Ligand	-	-	71.1
Water	30.24	50.0	52.3
r.m.s deviations			
Bond lengths (Å)	0.002	0.014	0.003
Bond angles (°)	0.45	2.05	0.52
Ramachandran Plot			
Favoured (%)	97.4	96.7	97.8
Outliers (%)	0.0	0.0	0.0

Supplementary Table 5. Structural similarity of Asgard RBs with other RBs, longins and profilins.

	Structure	PDB	Res/Å	Odin-RB_{LC7} RMSD/Å	Odin-RB_{LC7} Residues	Thor-RB RMSD/Å	Thor-RB Residues	MKD1-RB RMSD/Å	MKD1-RB Residues
MglB	*T. Thermophilus*	1j3w:C	1.5	1.47	70	1.63	110	1.61	109
	M. xanthus	6hjm:J	2.39	1.45	75	1.57	113	1.46	112
	S. avermitilis	3kye:A	2.15	1.33	77	1.55	116	1.6	112
Ragulator complex *H. Sapiens*	LAMTOR2	5y3a:B	2.9	1.84	75	1.65	108	1.54	110
	LAMTOR3	5y3a:C	2.9	2.31	72	1.96	106	2.31	106
	LAMTOR4	5y3a:D	2.9	1.71	71	2.11	84	1.85	81
	LAMTOR5	5y3a:E	2.9	1.77	71	1.81	85	1.69	84
Dynein intermediate chain light	*D. melanogaster RE64145p*	3l7h:A	1.95	1.41	78	1.65	83	1.32	82
TRAPP *S. cerevisiae*	TRS23	3cue:A	3.7	2.77	66	2.79	94	3.00	89
	BET5	3cue:C	3.7	2.46	68	2.50	94	2.60	86
Profilins	Odin	5zza:P	1.5	1.88	64	1.52	96	1.70	95
	Loki	5zzb:A	2.3	2.00	60	2.18	101	2.15	101
	A. thaliana	6iqj:A	1.9	1.94	64	2.59	102	2.34	95
	H. Sapiens	2pbd:P	1.5	1.85	60	2.68	97	2.68	100

Supplementary Table 6. X-ray data collection and refinement statistics for Thor-TRAPPC3.

	Thor-TRAPPC3<br>Thor (SMTZ1-45)<br>KXH75250.1<br>(PDB code 7YH3)	Thor-TRAPPC3<br>Thor (AB25)<br>OLS30461.1<br>(PDB code 7YH2)<br>(Native)	Thor-TRAPPC3<br>Thor (AB25)<br>OLS30461.1<br>(Peak λ)	Thor-TRAPPC3<br>Thor (AB25)<br>OLS30461.1<br>(Remote λ)
**Crystals**				
Crystallization conditions	100 mM HEPES<br>pH 7.5<br>250 mM $Mg(HCO_2)_2$<br>18% PEG 3350	100 mM HEPES<br>pH 7.5<br>250 mM $Mg(HCO_2)_2$<br>18% PEG 3350		
Lattice	$P2_1$	$P2_12_12_1$	$P2_12_12_1$	$P2_12_12_1$
a, b, c (Å)	68.4 62.1 69.3	63.3 68.5 72.7	63.4 68.3 72.8	63.4 68.3 72.8
α, β, γ (°)	90.0, 100.5, 90.0	90.0, 90.0, 90.0	90.0, 90.0, 90.0	90.0, 90.0, 90.0
**Data collection**				
Beamline	TPS 05A, NSRRC	TPS 05A, NSRRC	TPS 05A, NSRRC	TPS 05A, NSRRC
Wavelength (Å)	1.0	1.158	1.282	1.0
Resolution (Å)	20.0-1.70 (1.73-1.70)	50.0-1.91 (1.94-1.91)	50.0-1.96 (1.99-1.96)	50.0-1.96 (1.99-1.96)
R_{merge}	4.8 (30.1)	11.2 (42.6)	14.0 (63.8)	14.0 (61.9)
R_{meas}	5.6 (35.1)	11.7 (45.7)	14.6 (78.5)	14.7 (74.0)
R_{pim}	2.8 (17.7)	3.4 (16.2)	4.3 (44.8)	4.4 (39.6)
$I/\sigma(I)$	22.9 (2.2)	58.0 (6.5)	33.5 (1.3)	33.6 (1.5)
$CC_{1/2}$	(0.932)	(0.968)	(0.628)	(0.656)
Completeness (%)	94.9 (74.1)	99.7 (93.9)	95.6 (65.8)	99.7 (99.1)
Redundancy	3.8 (2.7)	11.7 (6.8)	9.2 (2.4)	6.6 (6.6)
**Refinement**				
Resolution (Å)	19.5-1.73 (1.76-1.70)	32.1-1.91 (1.98-1.91)		
No. reflections	51121 (2550)	25100 (2433)		
R_{work} / R_{free}	21.6/25.9 (29.4/35.9)	16.9/21.2 (18.8/24.2)		
No. atoms				
Protein	4974	2593		
Zn^{2+}	4	2		
Water	710	160		
B factors				
Protein	25.1	35.7		
Zn^{2+}	11.5	26.1		
Water	33.8	44.2		
r.m.s deviations				
Bond lengths (Å)	0.004	0.008		
Bond angles (°)	0.59	0.89		
Ramachandran Plot				
Favoured (%)	96.7	98.1		
Outliers (%)	0.17	0.0		

Supplementary Table 7. Structural similarity of Thor-TRAPPC3 with TRAPPC3/V4R structures.

			Thor-TRAPPC3 Thor (SMTZ1-45) 156 residues		Thor-TRAPPC3 Thor (AB25) 155 residues		
Structure	PDB	Res/Å	RMSD/Å	Residues	RMSD/Å	Residues	Ligand
**Bet3p/TRAPPC3**							
Yeast	7kmt:F	3.6	2.3	149	2.1	150	Palmitic acid
Drosophila	7b6x:A	3.7	2.4	151	2.3	152	-
Mouse	2j3t:A	2.4	2.2	148	2.1	149	Palmitic acid
**Trs31p/TRAPPC5**							
Yeast	7kmt:J	3.6	2.1	143	2.2	144	-
Drosophila	7b6x:F	3.7	2.4	146	2.2	146	-
**Trs33p/TRAPPC6**							
Yeast	7kmt:E	3.6	2.8	128	2.7	126	-
Drosophila	7b6x:B	3.7	3.2	121	3.3	128	-
Human	2j3t:B	2.4	2.8	135	2.8	136	-
**Aromatic sensor**							
MopR *Ac*	5kbi:A	2.9	3.3	131	3.3	133	Catechol/Zn^{2+}
PoxR *Cn*	5fs0:A	2.4	3.5	130	3.3	134	2,4-dichlorophenol/Zn^{2+}
**NO-binding heme-dependent sensor protein** H-NOX *So*	4u9k:A	2.5	3.3	126	3.2	125	Protoporphyrin IX/Zn^{2+}
**Soluble guanylate cyclase**							
GUCY1A Human	6jt0:A	4.0	3.4	115	3.3	113	-
GUCY1B Human	6jt0:B	4.0	3.1	124	3.3	125	Protoporphyrin IX
**Cellulose synthase sub. D**							
AxCeSD *Kx*	3a8e:A	3.0	3.5	120	3.2	120	beta-cellopentaose

Ac = *Acinetobacter calcoaceticus*, *Cn* = *Cupriavidus necator*, *Kx* = *Komagataeibacter xylinus*, *So* = *Shewanella oneidensis*, Res = Structure resolution
